# Atypical Beta Oscillatory Dynamics Are Related to Poor Procedural Learning in Children With Developmental Coordination Disorder

**DOI:** 10.1111/desc.70031

**Published:** 2025-05-21

**Authors:** Jarrad A. G. Lum, Kaila M. Hamilton, Li‐Ann Leow, Welber Marinovic, Ian Fuelscher, Pamela Barhoun, Talitha C. Ford, Aron T. Hill, Samaneh Nahravani, Melissa Kirkovski, Peter G. Enticott, Christian Hyde

**Affiliations:** ^1^ Cognitive Neuroscience Unit School of Psychology Deakin University Geelong Australia; ^2^ School of Arts & Humanities Edith Cowan University Joondalup Australia; ^3^ School of Psychology The University of Queensland St Lucia Australia; ^4^ School of Population Health, Discipline of Psychology Curtin University Perth Australia; ^5^ Faculty of Health, Arts & Design Swinburne University of Technology Hawthorn Australia

**Keywords:** alpha, beta, developmental coordination disorder (DCD), electroencephalography (EEG), neural oscillations, procedural learning, serial reaction time (SRT) task, theta

## Abstract

Procedural learning difficulties are commonly reported in children with developmental coordination disorder (DCD), yet the neural basis of this impairment remains unclear. This study addressed this gap by examining the correlation between cortical oscillatory activity and procedural learning of a sequence of finger movements in children with and without DCD. Participants were 19 children with DCD and 38 typically developing (TD) children, with a mean age of 10 years and 3 months. Children completed the Serial Reaction Time task, a standard measure of procedural learning, during which they unintentionally learned a sequence of finger movements. Electroencephalography (EEG) was continuously recorded as they performed the task. Behavioural analyses indicated poorer procedural learning in the DCD group compared to the TD group. EEG analyses revealed that beta activity over motor areas and theta/alpha activity over occipital areas were sensitive to procedural learning effects. Group differences were observed only in beta activity, with the DCD group showing reduced beta modulation relative to TD children. No significant group differences were found for theta or alpha activity. This study provides new evidence demonstrating an association between poor procedural learning and atypical beta oscillatory dynamics in DCD.

## 1 Introduction

Children with developmental coordination disorder (DCD) experience significant difficulties learning and executing coordinated movements (American Psychiatric Association 2013). These motor difficulties occur in the absence of intellectual, sensory or major neurological impairments, and negatively impact a child's capacity to perform common self‐maintenance tasks, including dressing, writing/typing and using cutlery (Magalhães et al. 2011; Summers et al. 2008; Van der Linde et al. 2015). The neural basis of these motor difficulties has yet to be fully established. To date, functional magnetic resonance imaging has been the dominant method for investigating real‐time brain functioning in DCD (Biotteau et al. 2016; Brown‐Lum and Zwicker 2015; Wilson et al. 2017). However, this approach lacks the temporal resolution necessary to capture rapidly changing neural dynamics, a fundamental mechanism for information processing and transfer in the brain (Buzsaki 2006; Logothetis and Pfeuffer 2004). To address this gap, the current study used electroencephalography (EEG) to investigate the neural oscillatory dynamics of motor learning in children with DCD.

**Summary**

- Children with developmental coordination disorder (DCD) showed reduced procedural learning compared to typically developing peers on the serial reaction time (SRT) task.
- EEG analyses revealed that beta oscillations over the motor cortex were sensitive to motor sequence learning.
- Children with DCD showed attenuated beta modulation, implicating disrupted bottom‐up sensorimotor processing in procedural learning impairments.
- Theta/alpha oscillations linked to top‐down visuo‐spatial attention were preserved in DCD, suggesting selective deficits in bottom‐up rather than top‐down neural mechanisms.

Procedural learning impairments have been proposed to underlie the motor learning and skill acquisition deficits observed in DCD (e.g., Clark and Lum 2017; Gheysen et al. 2011; Nicolson and Fawcett 2007). Although procedural learning is traditionally conceptualised as the acquisition and storage of new motor programs (Gabrieli 1998; Squire and Zola 1996; Willingham et al. 2002), alternative theories propose that coordinated movement patterns can emerge through processes of self‐organisation and adaptation, without reliance on stored motor commands (Chen et al. 2012; Turvey 1990; Wade and Kazeck 2018). The key distinction between these accounts being whether skill acquisition depends on the formation of stable, internalised representations that guide movement execution, or adaptation of movements to environmental and task constraints, without the need for stored representations. Common to both perspectives; however, is the view that successful skill acquisition ultimately results in fluid, coordinated movements, regardless of whether this occurs through the formation of internal motor programs or through processes of adaption.

Procedural learning enables individuals to execute motor skills without conscious awareness of the specific actions involved in the sequence. For instance, as typing skills improve, it becomes increasingly difficult to recall the exact finger movements used to type words and sentences (Salthouse 1986). Procedural learning in DCD has frequently been examined using Nissen and Bullemer's (1987) serial reaction time (SRT) task. In this task, a visual stimulus repeatedly appears in one of four predetermined spatial locations on a computer display. Participants press one of four buttons on a response pad that corresponds to the stimulus’ location as quickly as possible after its onset. Unbeknownst to the participants, the location of the stimulus follows a predetermined sequence, typically 8–12 elements in length. In both adult and paediatric control groups (e.g., Lum 2020; Lum and Kidd 2012), reaction times decrease (i.e., become faster) with repeated exposures to the sequence (for a review see Zwart et al. 2019). Crucially, reaction times increase (i.e., slow down) when the stimulus begins appearing randomly in one of the four positions at the end of the task. This increase in reaction times from sequence to random blocks of trials indicates sequence acquisition, as participants’ predictions conflict with the now random stimuli. If learning had not occurred, reaction times would continue to decrease or reach asymptote, following introduction of random trials (Janacsek and Nemeth 2013; Robertson 2007).

Poor procedural learning as assessed by the SRT task has been reported in DCD. In several studies, the increase in reaction times from sequence to random blocks was found to be attenuated in DCD (Bianco et al. 2024; Blais et al. 2021; Gheysen et al. 2011; Sinani et al. 2024; Van Dyck et al. 2023). This was most pronounced in a study by Gheysen et al. (2011) who presented the SRT task to 18 children with DCD and 20 age‐matched controls. In their version of the task, the children used the index and middle fingers from both hands to respond to the visual stimulus, thereby implicitly learning a sequence of finger movements. After multiple blocks of sequence trials, the control group's reaction times slowed down on the random block presented at the end of the task, while the reaction times of the children with DCD continued to decrease. Thus, the increase in reaction times from sequence to random blocks was smaller in the DCD group compared to controls. Notably, not all studies report poor procedural learning. In one study, non‐significant differences between DCD and controls on the SRT task can be explained by poor statistical power (Wilson et al. 2003). However, this does not appear to be the case in the study undertaken by Lejeune et al. (2013). In this research, the SRT task was presented to 17 children with and without DCD. To reduce demands on fine motor skills, children responded to a visual stimulus using a touchscreen. In this study, a sequence of limb movements was therefore learnt. The increase in reaction times from sequence to random blocks was found to be comparable between groups. These results suggest procedural learning may not be globally impaired in DCD; rather, the extent of the deficit may depend on the specific effector system engaged during the task.

To date, the neurobiology of procedural learning impairments in DCD has predominantly been interpreted in terms of a structural deficit affecting basal ganglia‐cortical circuitry (Bianco et al. 2024; Bo and Lee 2013; Nicolson and Fawcett 2007). Procedural learning on the SRT task is also associated with modulations in cortical oscillatory activity (Heideman et al. 2018; Lum et al. 2024; Lum et al. 2023; Zhuang et al. 1997). Cycles of synchronised and desynchronised firing by neuronal populations form the basis of information processing and transfer in the brain. In humans, neuronal populations typically synchronise at speeds of 1–4 Hz (delta), 4–7 Hz (theta), 7–12 Hz (alpha), 12–30 Hz (beta), and >30 Hz (gamma) (Buzsaki and Draguhn 2004). The postsynaptic electrical activity generated by cortical pyramidal neurons from this process results in rhythmic fluctuations or oscillations that are captured by scalp electrodes in the electroencephalogram (EEG) and magnetoencephalogram (MEG) (Cohen 2017). Generally, an increase in the number of neurons firing at the same frequency results in greater amplitude, or power (defined as amplitude squared), at a specific frequency band in the M/EEG (Pfurtscheller and Aranibar 1977). Modulations of oscillatory activity in the aforementioned frequency bands support higher‐order and sensorimotor functions (Ward 2003). There is growing evidence suggesting that neurodevelopmental disorders, including DCD, are associated with altered functioning in one or more of these frequency bands (e.g., Cainelli et al. 2023; Michelini et al. 2022; Neo et al. 2023; Wang et al. 2017). It has yet to be determined, however, whether impairments in cortical oscillatory activity affect procedural learning in DCD.

Much is now known about changes in cortical oscillatory power during procedural learning on the SRT task, at least in adult samples. First, beta power, recorded from electrodes/sensors over the motor areas, decreases as the sequence of finger movements is unknowingly repeated, then increases when random movements are introduced (Lum et al. 2024; Tzvi et al. 2016; Zhuang et al. 1997). This decrease in beta power might be related to encoding the motor sequence. In support, Meissner et al. (2018) found that adults with Parkinson's disease not only demonstrated poorer procedural learning than controls, but the difference in beta power between sequence and random movements was also smaller in the Parkinson's group.

Decreases in beta power indicate active processing (Pfurtscheller et al. 2003). For example, the execution of finger or hand movements is associated with a reduction in beta power over regions contralateral to the motor response (Pfurtscheller 1992). This decrease occurs because functional networks needed to complete the task desynchronise from broader resting‐state networks, to process relevant information. Once completed, the previously activated network then re‐synchronises with resting‐state networks, leading to an increase in beta power (Pfurtscheller and Da Silva 1999). Beta‐band oscillations play a broader role in supporting higher‐order functions beyond movement execution (e.g., Schmidt et al. 2019; Weiss and Mueller 2012). Engel and Fries (2010) suggest beta desynchronisation enables incoming sensory information (e.g., from finger movements) to modify functional networks, which forms the basis of bottom‐up learning. In the context of the SRT task, Lum et al. (2024) suggested beta desynchronisation facilitates the discovery of correlated finger movements and representation of the sequence by the motor system. Conversely, beta synchronisation (i.e., an increase in beta power) may serve to protect previously learnt information to guide future responses. Increases in beta power during random trials on the SRT task may help preserve the newly acquired motor sequence from interfering information.

Second, theta power recorded outside motor areas also decreases as the sequence is repeated but increases with random movements on the SRT task (Lu et al. 2023; Lum et al. 2024; Lum et al. 2023). Increases in theta power are typically observed on tasks requiring active monitoring of incoming information or the encoding of information into declarative or working memory (Eisma et al. 2021; Jensen and Tesche 2002; Van Der Cruijsen et al. 2021). Decreases in theta power during sequence trials likely indicate disengagement of top‐down attentional processing from the learning process. This may facilitate procedural learning. According to several models, procedural learning is optimally achieved through the bottom‐up discovery of information from sensory input (Foerde et al. 2006; Janacsek et al. 2012; Smalle et al. 2022). The deployment of top‐down processes may hinder this process by diverting neural resources to seemingly task‐relevant information (Nemeth et al. 2013; Pascual‐Leone et al. 1996; Smalle et al. 2022). For example, on the SRT task, an attempt to encode motor or visual information into working memory or declarative memory may reduce the capacity of the motor system from detecting the hidden sequence of finger movements (e.g., Brown and Robertson 2007). Poor procedural learning in DCD may also relate to difficulties disengaging the theta‐mediated top‐down system.

### 1.1 Aims and Hypotheses

To our knowledge, no study has examined oscillatory activity associated with procedural learning in DCD. Based on the studies reviewed above, atypical beta and/or theta activity may reflect a disruption to this type of learning in the disorder. The aim of the current study was to examine cortical oscillatory activity associated with procedural learning in children with and without DCD. The study included 19 children with DCD and 38 typically developing (TD) children, all aged around 10 years. The children were presented with the SRT task in which they unintentionally learnt a sequence of finger movements. At the behavioural level, we predicted that the DCD group would show poorer procedural learning compared to TD children. As children completed the SRT task, we also examined changes in oscillatory power using EEG. For TD children, we predicted that repeating a sequence of movements on the task would be associated with lower theta and beta power, relative to random movements, replicating findings from studies undertaken with healthy adults (e.g., Lum et al. 2024). For children with DCD, we hypothesised that poorer procedural learning would co‐occur with an atypical beta and/or theta response. Specifically, on the task, we predicted that the increase in beta and theta power from sequence to random blocks would be attenuated in the DCD group. To further investigate the relationship between oscillatory power and procedural learning, we conducted a series of correlation analyses examining the association between oscillatory power and manual reaction times. As noted above, beta and theta desynchronisation have been suggested to promote procedural learning. If this is the case, faster reaction times on the SRT task might be associated with lower theta and beta power.

## 2 Method

### 2.1 Participants

A total of 19 children with DCD and 38 TD children participated in the study. Demographic information is presented in Table 1. One child with DCD had a clinical diagnosis of ADHD but was not taking medication at the time of testing. Children were recruited via social media advertisements and through occupational therapists. Participants were drawn from an ongoing study (Bianco et al. 2024) in which relatively noise‐free EEG data were obtained during the SRT task. This research was conducted with the approval of the Deakin University Human Research Ethics Committee and adhered to the principles of the Declaration of Helsinki (World Medical Association 2024). Informed written consent was obtained from the parents of all participants, and assent was obtained from the children. Families received a $30 AUD shopping voucher for their participation. No a priori sample size calculation was conducted. Instead, the sample size was determined by the number of children who met the criteria for either the DCD or control group (as described below) and from whom we could obtain relatively noise‐free EEG data.

**TABLE 1:** Demographic and clinical characteristics of children with developmental coordination disorder (DCD) and typically developing (TD) controls.

Variable	DCD		TD		*p* value
	M or %	SD	M or %	SD	
% Female	52.6%	—	47.4%	—	0.708^b^
% Right‐handed	84.2%	—	89.5%	—	0.568^b^
Age (years; months)	10;4	2;6	10;4	2;1	0.993^c^
Age range	(6;3–14;4)		(6;6–14;10)		
BOT‐2 SF^a^	36.1 (9th %ile)	3.1	50.8 (53rd %ile)	7.8	<0.001^c^
DCD‐Q (total score)^a^	36.1 (8th %ile)	9.5	63.3 (90th %ile)	8.7	<0.001^c^
ADHD‐RS IV (total raw score)	17.5	13.0	10.4	10.5	0.035
WASI‐2 matrix reasoning^a^	46.8	12.9	56.8	10.8	0.019^c^
WASI‐2 vocabulary^a^	50.0	12.6	54.8	8.7	0.263^c^

Abbreviations: ADHD RS‐IV, Attention Deficit Hyperactivity Disorder Rating Scale; BOT‐2 SF, Bruininks–Oseretsky Test of Motor Proficiency 2nd Edition Short Form; DCD‐Q, Developmental Coordination Disorder Questionnaire; WASI‐2, Wechsler Abbreviated Scale of Intelligence‐2^nd^ Edition.

^a^ Data shown in the table are T‐scores (i.e., mean of 50 and standard deviation of 10).

^b^ Fischer's exact test evaluated differences in frequencies.

^c^ Mann–Whitey *U* tests evaluated differences between groups.

All children in the DCD group met the DSM‐5 criteria for the disorder. Specifically, they exhibited motor skills below age‐appropriate levels (Criterion A), as indicated by performance below the 15th percentile on the short‐form version of the Bruininks–Oseretsky Test of Motor Proficiency, Second Edition (BOT‐2 SF; Bruininks and Bruininks 2005). The BOT‐2 SF assesses both fine and gross motor skills. An overall score that combines these two domains was used to assess motor functioning in the sample. Second, motor impairment was determined to negatively impact daily activities (Criterion B), assessed using the Developmental Coordination Disorder Questionnaire (DCD‐Q; Wilson et al. 2009). This is a parental report that evaluates the extent to which motor impairments affect daily functioning. In the absence of Australian norms for the DCD‐Q, functional motor impairment was identified using normative data from previous work with TD children (Bianco et al. 2024). Based on this sample, 95% confidence intervals for DCD‐Q scores were calculated for three age groups: 5–7 years (CI: 59.75 ± 3.48), 8–9 years (CI: 64.00 ± 4.44), and 10–14 years (CI: 65.05 ± 4.02). All children with DCD in the current study scored below the lower bound of the 95% confidence interval for their respective age group. Third, since participants were children, we could establish that the motor difficulties emerged during childhood (Criterion C). Finally, motor difficulties were not attributable to any pre‐existing medical, sensory, or intellectual disabilities (Criterion D). This criterion was assessed based on medical records reported by the children's parents. Children in the TD group demonstrated age‐appropriate motor skills, scoring above the 16th percentile on both the BOT‐2 and DCD‐Q.

General cognitive functioning was assessed using the Matrix Reasoning and Vocabulary subtests from the Wechsler Abbreviated Scale of Intelligence, 2nd Edition (WASI‐II; Wechsler 2011). The Matrix Reasoning subtest assesses visuo‐spatial reasoning, and the Vocabulary subtest assesses semantic knowledge. Both subtests were scored using T‐scores. Handedness was assessed through parental reports. Also, considering the frequent co‐occurrence of DCD and ADHD, we assessed attentional difficulties in our sample using the ADHD Rating Scale‐IV RS (ADHD‐RS‐IV; DuPaul et al. 1998). This scale evaluates overall ADHD symptom severity. In the present study, we used raw scores, which can range from 0 to 54, with higher values reflecting increased frequency and severity of ADHD‐related behaviours.

Table 1 provides summary statistics from standardised tests, along with the age, gender, and handedness distributions for each group. The table also includes results from statistical tests comparing the groups on all variables. Significant group differences were observed on the DCD‐Q and BOT‐2. Furthermore, the DCD group evidenced significantly higher raw scores from the ADHD‐RS‐IV, indicating elevated levels of attentional difficulties and hyperactivity symptoms compared to the TD group. This result is to be expected since attentional and motor impairments often co‐occur (Sergeant et al. 2006). No significant differences were found between the groups for age, gender, handedness, or WASI‐II Vocabulary subtest scores. The DCD group, however, scored significantly lower on the WASI‐II Matrix Reasoning subtest than the TD group, likely reflecting visuo‐spatial difficulties associated with the disorder (Wilson and McKenzie 1998).

### 2.2 Materials

#### 2.2.1 Serial Reaction Time Task

**FIGURE 1:**
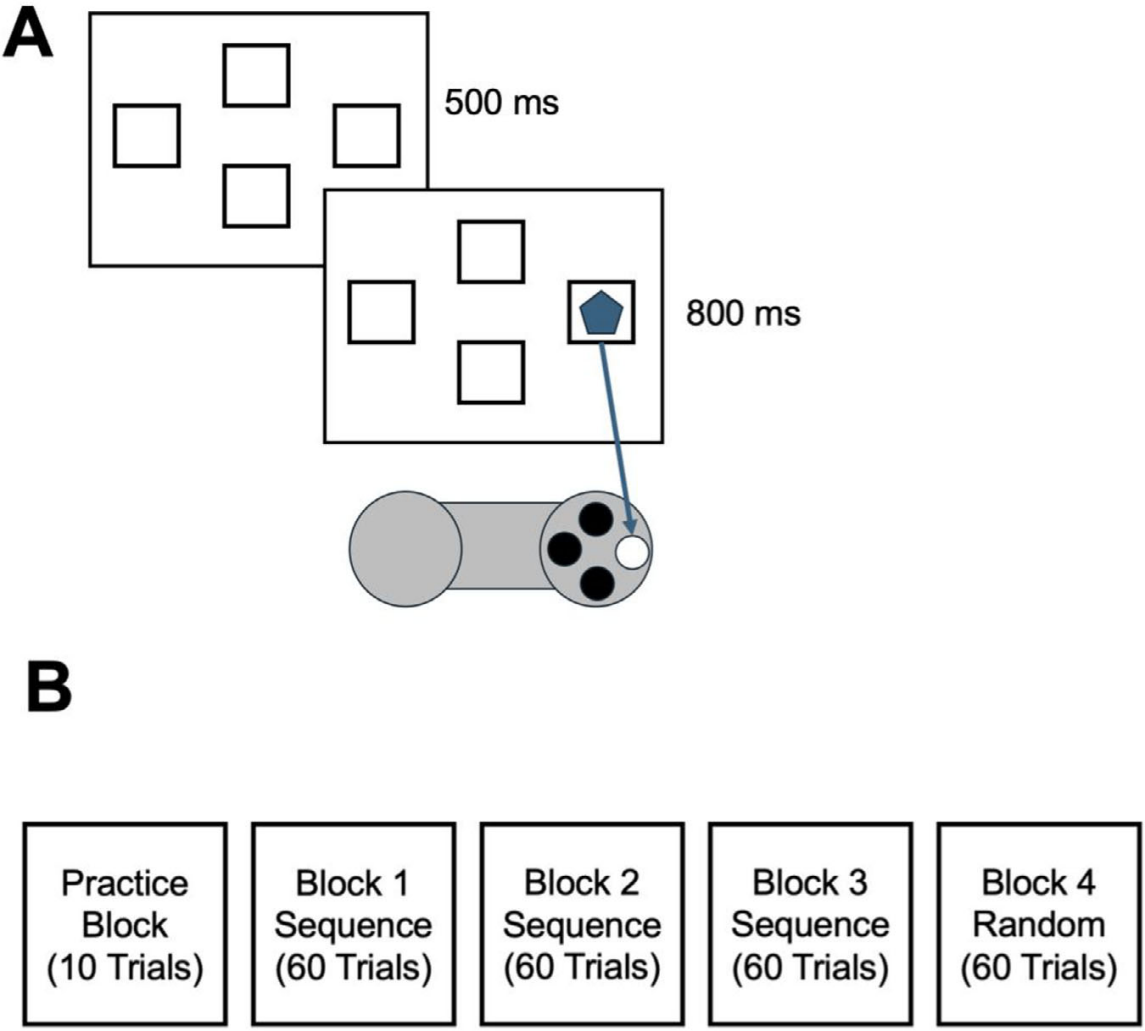
Overview of the SRT task. Panel A presents an overview of a single trial and response panel. Panel B shows the order blocks were presented along with the number of trials.

Procedural learning was assessed using a version of the SRT task previously employed with children (Lum and Kidd 2012). An overview of the task is presented in Figure 1. In this version, a visual stimulus appeared on a computer display in one of four locations, arranged in a diamond configuration. Children were instructed to press one of the four buttons on the gamepad, that were also arranged in a diamond configuration to match the location of the visual stimulus. All children operated the gamepad using their right thumb (see Panel A of Figure 1). The requirement for all children to use their right hand to generate a motor response was based on research showing that EEG recordings are sensitive to lateralisation effects, particularly in the motor domain (e.g., Pfurtscheller 1992). By standardising hand use, we aimed to increase the sensitivity of our analyses in detecting procedural learning‐related beta power modulations at motor electrodes.

The SRT task consisted of four blocks of 60 trials (see Panel B of Figure 1). Each trial began with the computer display presenting four empty boxes, outlined in black and arranged in a diamond configuration, for 500 ms. A visual stimulus, consisting of a randomly selected shape (e.g., circles or polygons), then appeared for 800 ms. During this time, children were required to provide a manual response. The visual stimulus remained on screen for the entire 800 ms, regardless of response timing. For example, if a response was made 300 ms after stimulus onset, the stimulus would still be displayed for the remaining 500 ms. This approach ensured that the duration of all trials and task length was identical for all participants.

Children were unaware that during Blocks 1–3, the trial‐to‐trial location of the visual stimulus followed a predetermined 10‐element sequence. Labelling the left‐most position of the diamond configuration as 1, top‐most as 2, right‐most as 3, and bottom‐most as 4, the sequence was: 3‐4‐1‐2‐4‐1‐3‐4‐2‐1. In Block 4, however, the visual stimulus appeared randomly in one of the four spatial locations, adhering to the following constraints: First, the stimulus did not appear in the same location on consecutive trials. Second, the frequency of the stimulus appearing in each location in Block 4 was the same as in Blocks 1–3. For example, in each of Blocks 1–3, the visual stimulus appeared in Position 1 a total of 18 times. In Block 4, the visual stimulus also appeared in Position 1 a total of 18 times. Third, the first‐order transitional probabilities in Block 4 were the same as in Blocks 1–3. For example, if the visual stimulus appeared in Position 1 on the sequence blocks, there was 33% probability it would appear in Position 2 on the next trial, this probability remained the same in Block 4. These constraints ensured that any observed differences between the sequence and random blocks reflected sequence learning rather than stimulus pair learning. The task was administered using E‐Prime 2 software (Psychology Software Tools, Pittsburgh, PA, USA).

Both accuracy and reaction times were recorded as children responded to the visual stimulus. However, only reaction times were used to investigate procedural learning in the DCD and TD groups, as this was the primary outcome variable of interest from the SRT task. A correct response was defined as pressing the button on the gamepad corresponding to the location of the visual stimulus on the screen. An incorrect response was defined as pressing the wrong button or responding after the 800 ms window. Accuracy for both groups was near ceiling. The mean proportion of correct responses for the DCD group was 0.870 (*SD *= 0.149) and for the TD group 0.922 (*SD* = 0.092). A Mann–Whitney *U* test indicated that this difference was not statistically significant (*p *= 0.173). Reaction times were defined as the time (in ms) between stimulus onset and the manual response. For each child, mean reaction times were computed for each block, and these data were used to assess differences in procedural learning between the DCD and TD groups. Only reaction times from correct responses were included in the analyses.

### 2.3 Procedure

Each child was individually tested in a laboratory setting. An elastic EEG cap (EasyCap, GmbH, Germany) was first fitted to each child's head (see section ‘Electroencephalography’ for details). EEG data were acquired using 23 Ag/AgCl electrodes embedded in the cap and positioned according to the 10/20 system. Electrodes were placed at the following locations: Fp1, Fp2, F9, F7, F3, AFz, F10, Fz, F4, F8, M1, T7, C3, Cz, C4, T8, M2, P7, P3, Pz, P4, P8, O1, and O2. Electrode impedances were reduced to below 10 kΩ before starting the EEG recording. Children were first introduced to the gamepad and provided with instructions for the SRT task. A series of 10 practice trials followed, during which the visual stimulus appeared randomly in one of the four spatial locations on the computer display, and children were asked to press the corresponding button on the gamepad. These practice trials ensured that children understood the goal of the task. All participants achieved at least 80% accuracy on the practice items. After practice, children proceeded to Blocks 1–4 of the SRT task.

### 2.4 Electroencephalography

#### 2.4.1 Data Acquisition

EEG data were continuously recorded as the children performed the SRT task using a tMSI RefA amplifier (Twente Medical Systems International, The Netherlands) and Polybench software (Version 1.30; Twente Medical Systems International, The Netherlands). Data were acquired at a sampling rate of 2048 Hz, utilising a common average reference, with electrode AFz serving as the ground. Event codes were inserted during acquisition to mark stimulus onset, manual responses and response accuracy.

#### 2.4.2 Pre‐Processing

EEG data were pre‐processed using EEGLAB (Delorme and Makeig 2004) and the Reduction of Electroencephalographic Artifacts (RELAX) (Bailey, Biabani, et al. 2023; Bailey, Hill, et al. 2023) toolboxes run in MATLAB (Version #2023A). First, the data were downsampled to 250 Hz. The RELAX pre‐processing pipeline was applied using default settings. The RELAX pipeline applies a 0.25–80 Hz bandpass filter and corrects the signal for oculomotor, line noise, muscle and other artifacts using Multiple Wiener Filtering, wavelet‐enhanced Independent Component Analysis, and ICLabel for artifact identification. The pipeline also includes the ‘findNoisyChannels’ function from the ‘PREP’ preprocessing pipeline toolbox (Bigdely‐Shamlo et al. 2015) to identify and interpolate excessively noisy channels. After pre‐processing, the data were re‐referenced to the average of the left and right mastoids. Electrodes Fp1, Fp2, F9, and F10, which were used to capture oculomotor activity, were excluded from further analyses. Finally, epochs were created from the continuous EEG data, spanning ±2000 ms from stimulus onset. Although the final analyses focused on oscillatory activity during the stimulus presentation window (i.e., 800 ms post‐stimulus onset), data from adjacent trials were initially included in the epochs to avoid edge effects from the time‐frequency analyses. Similar to the analyses of reaction time data, only epochs associated with a correct manual response were used in the analyses.

#### 2.4.3 Time‐Frequency Analysis

The time‐series data from each epoch and electrode were submitted to time‐frequency decomposition using complex Morlet wavelets. The wavelets were defined as $e^{i2\pi ft}e^{-t^{2}/\left(2\sigma^{2}\right)}$, with frequency values (*f*) ranging from 1 to 35 Hz in 90 linearly spaced steps. The wavelet width (σ  =  *n*/[2π*f*]), defined in cycles (*n*), ranged from 2 to 15 logarithmically spaced steps. These parameters provided suitable temporal and frequency resolution across the frequency ranges examined. The initial result of the time‐frequency decomposition was oscillatory activity measured in terms of power (µV^2^), computed at all frequencies between 1 and 35 Hz for each time point. Next, epoch‐level power data were averaged separately for each block of trials from the task, resulting in four sets of time‐frequency data per child (i.e., time‐frequency data for Blocks 1, 2, 3 and 4). The averaged data were then trimmed to focus on brain activity from −500 to 800 ms after stimulus onset, covering the period when the stimulus appeared and a response was required. The trimmed data were then decibel (dB) baseline‐corrected to the average of −500 to −100 ms period. In a final step the baseline‐corrected time‐frequency data were downsampled to 50 Hz. This approach preserves the temporal resolution of the time‐frequency data while reducing the computational time required for data analysis (Cohen 2014). Time‐frequency analysis was undertaken in MATLAB using scripts from Cohen (2014).

### 2.5 Data Analyses

#### 2.5.1 Reaction Time Data

**FIGURE 2:**
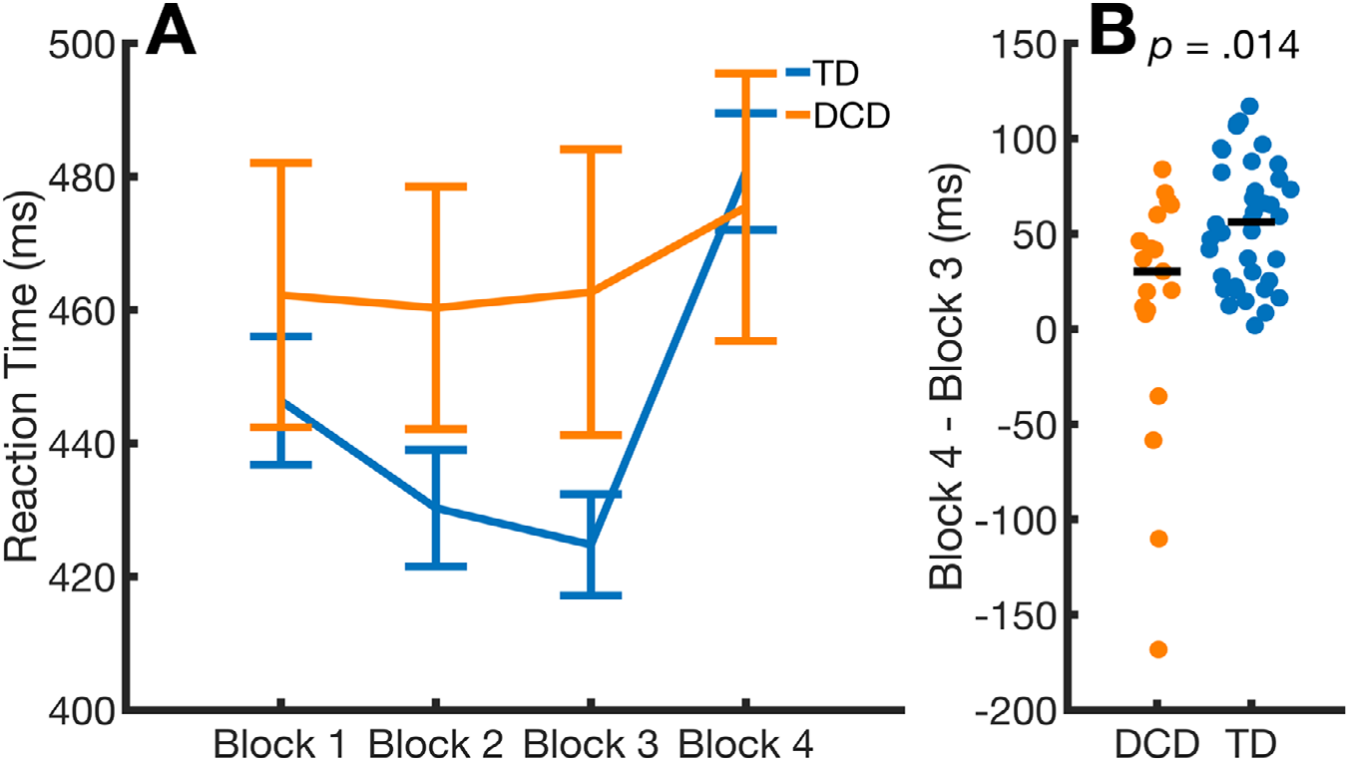
Summary of reaction time data from the SRT task by group. Panel A shows mean reaction times by group and block, with error bars representing standard errors. Panel B shows individual data points for the measure of procedural learning, computed as the difference between Block 4 and Block 3 reaction times, with the median value indicated by a horizontal line.

Procedural learning was assessed by comparing reaction times between the final sequence block (Block 3) and the subsequent random block (Block 4), following the standard approach in the literature (Robertson 2007). If sequence learning occurred, a significant increase in reaction times is expected from Block 3 to Block 4. To compare procedural learning between the DCD and TD groups, the difference in reaction times between Block 3 and Block 4 was calculated for each child using the formula: Block 4–Block 3. Positive values from this calculation would indicate slower reaction times following the introduction of the random block, suggesting greater sensitivity to the repeating sequence. It was hypothesised that this difference score would be smaller in the DCD group compared to the TD group, assuming procedural learning is impaired in the disorder. Non‐parametric tests were used for all analyses of reaction time, as the data did not meet the assumptions of parametric testing and to reduce the influence of extreme data points on the analyses (e.g., See Panel B of Figure 2). First, Wilcoxon Signed‐Ranks Tests were used to test whether the increase in reaction times from Block 3 to Block 4 was significant. This analysis was undertaken separately for each group. Second, a Mann‐Whitney U test evaluated whether the difference in reaction times between Block 3 and 4 differed between the groups. Thus, reaction time data were analysed by three statistical tests. To control for an inflated Type I error rate due to multiple comparisons, the Benjamini and Hochberg (1995) false discovery rate procedure was applied to the set of three *p* values.

#### 2.5.2 Time‐Frequency Data

Two sets of analyses were conducted to examine oscillatory activity associated with procedural learning in the sample. First, permutation testing with cluster correction (Cohen 2014; Maris and Oostenveld 2007; Nichols and Holmes 2002) was used to identify oscillatory activity sensitive to sequence learning effects. Specifically, we tested for significant differences in power between Block 3 (the final sequence block) and Block 4 (the random block). The rationale for contrasting these two blocks was to isolate brain activity sensitive to the repeating sequence, from brain activity related to generating a manual response to the visual stimulus. This contrast is commonly used in EEG (Lum et al. 2024), fMRI (Janacsek et al. 2020) and MEG (Meissner et al. 2018) neuroimaging SRT task research. Permutation testing enables thousands of univariate tests while maintaining an alpha level of 0.05. This approach was necessary because we were testing for significant differences in power across multiple electrodes (*n* = 17), frequencies (1–35 Hz; *n* = 90) and time points (0–800 ms at 50 Hz; *n* = 33). To improve statistical power, permutation testing was undertaken by combining data from the DCD and TD groups.

The goal of permutation testing was to derive an empirical null distribution of the largest cluster sizes in terms of pixel counts we would expect by chance, assuming no reliable difference between Block 3 and Block 4. This was achieved by shuffling or randomising Block 3 and Block 4 labels across participants. Next, *t*‐values were computed at each pixel and electrode in the permuted data, and these were thresholded using an alpha value of 0.05 (two‐tailed) to identify significant clusters. A cluster was defined as two or more adjacent significant pixels. The size of the largest cluster across all electrodes was extracted as one data point in the empirical null distribution. This process of shuffling labels, computing *t*‐values, and extracting the largest cluster size was repeated 10,000 times (i.e., 10,000 permutations were performed). In the non‐permuted (or ‘real’) data, *t*‐maps were generated, and significant clusters of power data were identified by thresholding pixels in the time‐frequency matrices at all electrodes. A cluster in the real data was considered statistically significant if its size (i.e., number of pixels) exceeded the 95th percentile of the largest clusters from the empirical null distribution, corresponding to *p* < 0.05. The permutation testing was implemented using modified scripts from Cohen (2014).

The second set of analyses tested for differences in oscillatory power between the TD and DCD groups. For this analysis, power from each significant cluster identified by the permutation testing was averaged separately for each block and participant. Group differences in procedural learning‐related oscillatory power were assessed using the same approach adopted for reaction time data. That is, for each cluster Wilcoxon Signed‐Ranks Tests evaluated, separately for each group, whether the increase in power from Block 3 to Block 4 was significant. Then, a Mann–Whitney *U* test evaluated whether the difference in average power between Block 3 and 4 differed between the groups. For each cluster, three statistical tests were therefore undertaken. As with the reaction time data, *p* values were corrected using the Benjamini and Hochberg (1995) false discovery rate procedure.

## 3 Results

### 3.1 Behavioural Data

The initial analyses examined procedural learning on the SRT task across DCD and TD groups. Group‐based reaction time data from the task are presented in Figure 2. Panel A of Figure 2 shows the mean reaction times (and standard errors) reported by block and group. This panel illustrates the expected learning trend, with reaction times decreasing across sequence blocks (Blocks 1–3) and increasing on the random block (Block 4). However, this modulation in reaction times is less pronounced in the DCD group. Results from the Wilcoxon rank‐sum test indicate that the increase in reaction times from Block 3 to Block 4 was significant for the TD group (*z* = 5.373, *p*_FDR Corrected_ < 0.001, *r* = 0.872), but not for the DCD group (*z* = 1.569, *p*_FDR Corrected_ = 0.117, *r* = 0.360). Panel B of Figure 2 presents swarm plots showing individual data points for the procedural learning measure, calculated as ‘Block 4 reaction times’ minus ‘Block 3 reaction times’. Positive values from this calculation indicate greater sensitivity to the sequence, as reaction times slowed following the introduction of the random block. Results from the Mann–Whitney *U* test revealed that the increase in reaction times from Block 3 to Block 4 was significantly smaller in the DCD group compared to the TD group (*z* = 2.607, *p*_FDR Corrected_ = 0.014, *r* = 0.598). This difference does not appear to be related to group differences in ADHD‐RS, WASI‐II, or handedness. The ‘Block 4–Block 3’ procedural learning measure was not found to be significantly correlated with WASI‐II Vocabulary (*ρ* = 0.133, *p* = 0.322), WASI‐II Matrix Reasoning (*ρ* = 0.143, *p* = 0.290), ADHD‐RS IV (*ρ* = −0.069, *p* = 0.610) or handedness (*ρ* = −0.042, *p* = 0.755; right‐handedness coded as 1 in the analyses).

### 3.2 Time‐Frequency Data

#### 3.2.1 Results From Permutation Testing

**FIGURE 3:**
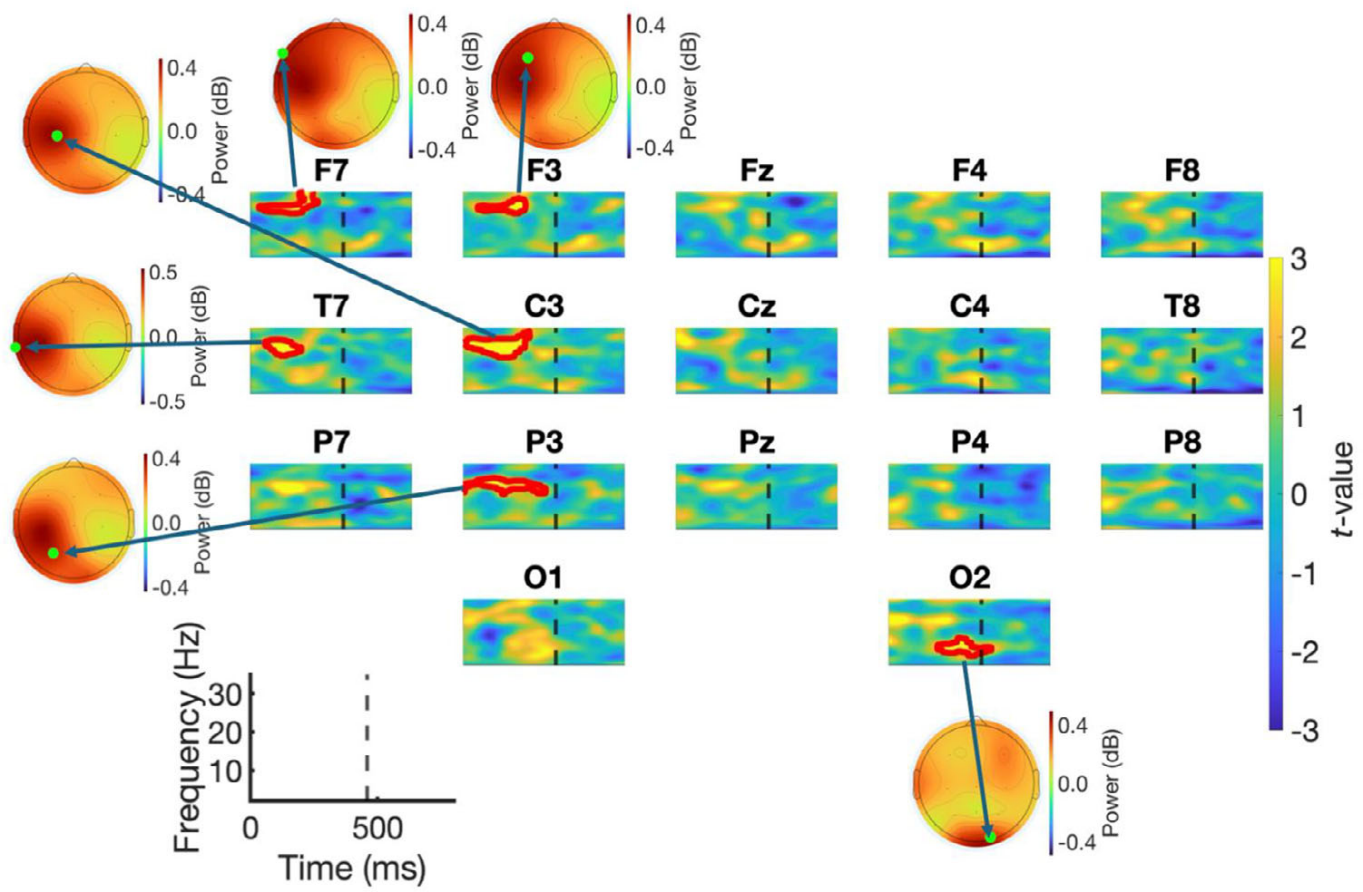
*t*‐maps contrasting baseline corrected power (dB) between Blocks 3 and 4. Lighter colours (yellow) indicate power was higher on Block 4 compared to Block 3. Darker colours (blue) indicate power was higher on Block 3 compared to Block 4. Contour lines (red) indicate clusters of pixels found to be significant from permutation testing. Also presented in the figure are topographic plots showing the distribution in power (dB) of each significant cluster.

Figure 3 displays time‐frequency *t*‐maps for each electrode, showing power between Blocks 3 and 4 of the SRT task. Data from both groups were combined for this analysis. In each time‐frequency plot, lighter colours (yellow) indicate data points where power was higher in Block 4 compared to Block 3, while darker colours (blue) indicate data points where power was lower in Block 4 than in Block 3. In each plot, time zero represents stimulus onset, and the dashed vertical line marks the average reaction time across Blocks 3 and 4, indicating the typical timing of manual responses. Red contour lines show clusters of pixels that were statistically significant according to permutation testing. Significant clusters were identified at electrodes F7 (*p* = 0.020), F3 (*p* = 0.042), T7 (*p* = 0.035), C3 (*p* < 0.001), P3 (*p* = 0.009) and O2 (*p* = 0.024). For all clusters, power was higher in Block 4 (random block) compared to Block 3 (sequence block). Also shown in Figure 3 are topographical distributions of power (dB, baseline‐corrected) for each statistically significant cluster. Table 2 presents the frequency and latency range of the significant clusters for each electrode. Permutation testing identified two main patterns of oscillatory activity sensitive to sequence learning effects. First, beta activity was observed over the left hemisphere, predominantly centred at electrode C3, with latency ranges occurring before the manual response in the beta band (>15 Hz). Second, theta/alpha activity was found at electrode O2 also appearing before a manual response was made.

**TABLE 2:** Summary table showing the latency range, frequency range/bands along with observed *p* value for each cluster found to be statistically significant.

Electrode	Latency range (ms)			Frequency range (Hz)			Frequency band/s	*p* value for cluster
F7	50	—	325	24.3	—	35	Beta	0.020
F3	75	—	300	24.0	—	31.2	Beta	0.042
T7	75	—	250	21.2	—	28.1	Beta	0.035
C3	0	—	325	20.1	—	32.7	Beta	<0.001
P3	0	—	400	20.1	—	27.7	Beta	0.009
O2	250	—	500	6.3	—	15.5	Theta/Alpha	0.024

*Note*: Each row in this table corresponds to a cluster (marked by the red contour line) presented in Figure 3.

#### 3.2.2 Oscillatory Dynamics of Procedural Learning in DCD and TD Groups

**FIGURE 4:**
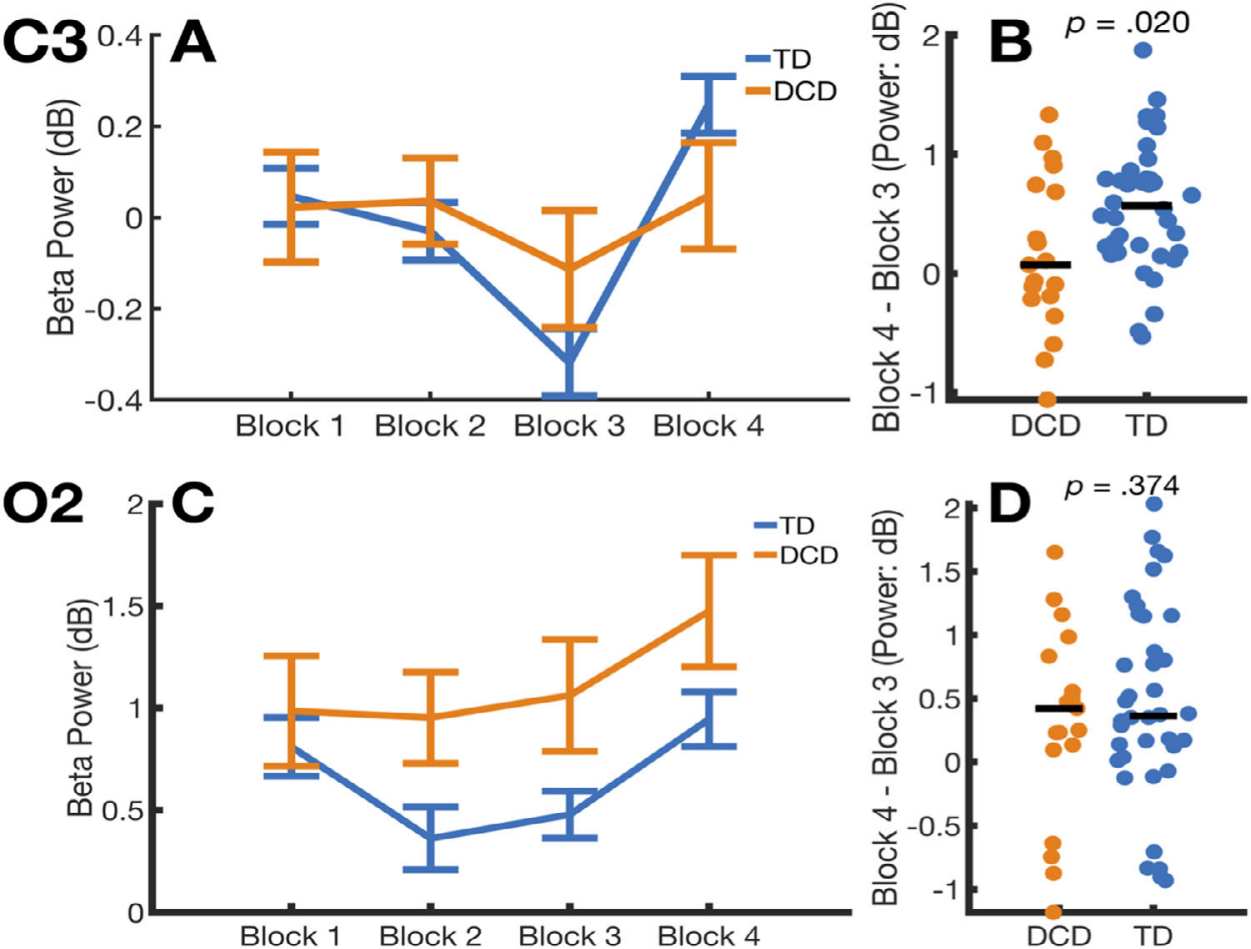
Changes in oscillatory power over the course of the SRT task. Panels A and C show mean power reported by group and block. In these panels, error bars show standard error. Panels B and D show individual data points for the measure of procedural learning.

The next set of analyses examined differences between the DCD and TD groups in theta/alpha and beta clusters identified by permutation testing. We specifically assessed whether the significant cluster of beta power at electrode C3 and cluster of theta/alpha power at electrode O2 were sensitive to sequence learning effects and differed between groups. The decision to focus on electrode C3 was based on visual inspection of the topographical plots in Figure 3, which indicated that sequence learning effects were most pronounced at this site. For these analyses, we averaged power for each cluster separately for each child, block and electrode. Panels A and C in Figure 4 show mean cluster power by group and block observed at electrode C3 and O2, respectively. The general trend in Figure 4 indicates that for both groups, power at each of the studied electrodes declined across repeated sequence blocks (Blocks 1–3) and then increased with the introduction of random stimulus presentations (Block 4). Panels B and D in Figure 4 show swarm plots of individual data points for procedural learning, computed as the difference between Block 4 and Block 3 power. Positive values on this index indicate higher power on the random block compared to the sequence block.

At electrode C3, the increase in power from Block 3 to Block 4 (Panel A, Figure 4) was significant for the TD group (*z* = 4.648, *p*_FDR Corrected_ < 0.001, *r* = 0.754), but not for the DCD group (*z* = 0.885, *p*_FDR Corrected_ = 0.376, *r* = 0.203). An analysis directly comparing this change in beta power across these two blocks between the DCD and TD groups was significant (*z* = 2.328, *p*_FDR Corrected_ = 0.020, *r* = 0.534; Panel B, Figure 4). Similar to reaction time data from the SRT task, the difference in beta modulation was not associated with scores from the ADHD‐RS IV, WASI‐2 or handedness. Specifically, the difference in beta power between Block 3 to Block 4 was not significantly correlated with WASI‐2 Vocabulary (*ρ* = 0.071, *p* = 0.601), WASI‐2 Matrix Reasoning (*ρ* = 0.189, *p* = 0.159), ADHD‐RS IV (*ρ* = −0.165, *p* = 0.300) and handedness (*ρ* = 0.170, *p* = 0.206; right handedness coded as 1 in the analyses).

At electrode O2, the increase in power from Block 3 to Block 4 (Panel C, Figure 4) was significant for the TD group (*z* = 3.401, *p*_FDR Corrected_ = 0.002, *r* = 0.552) and also for the DCD group (*z* = 2.213 *p*_FDR Corrected_ = 0.040, *r* = 0.508). The between‐group difference in the increase from Block 3 to Block 4 was not significant (*z* = 0.990, *p*_FDR Corrected_ = 0.322, *r* = 0.227; Panel D, Figure 4). The difference in alpha/beta power between the blocks was also not found to be significantly correlated with WASI‐2 Vocabulary (*ρ* = 0.068, *p* = 0.461), WASI‐2 Matrix Reasoning (*ρ* = 0.036, *p* = 0.700), ADHD‐RS IV (*ρ* = 0.042, *p* = 0.649) and handedness (*ρ* = 0.021, *p* = 0.846).

#### 3.2.3 Correlation Analyses Between Reaction Times, Theta/Alpha and Beta Power

**FIGURE 5:**
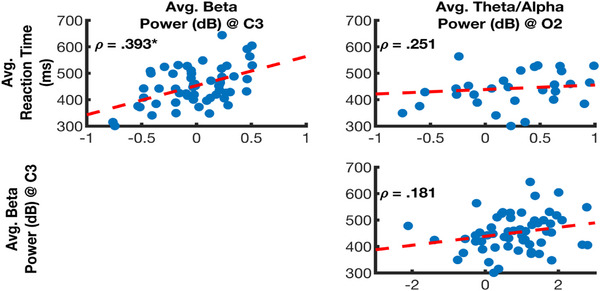
Correlations (Spearman's *ρ*) between oscillatory power and reaction times. Correlation computed by combining DCD and TD samples (total *n* = 57). *p < 0.05 after applying FDR correction.

The final set of analyses examined whether significant clusters at electrodes C3 (beta power), and O2 (theta/alpha power) correlated with individual differences in reaction times on the SRT task. We also examined power‐to‐power correlations between electrodes; specifically, whether beta power at electrode C3 correlated with theta/alpha power at O2. For these analyses, reaction times along with data from Panels A and C in Figure 4 were averaged across all blocks and for each child. Spearman's *ρ* was used to examine correlations between variables to minimise the influence of extreme data points. Benjamini and Hochberg's (1995) false discovery rate procedure was applied to the set of three *p* values. Finally, to improve statistical power, data from both groups were combined, with separate group correlations provided in the supporting information (see Table S1; computing correlations separately for each group generally produced similar patterns to those in the combined data set). Figure 5 presents a matrix of scatterplots for the studied variables. Beta activity at electrode C3 was found to be positively correlated with reaction times (*p*_FDR Corrected_ < 0.001), indicating that faster reaction times were associated with lower levels of beta power. All other correlations were non‐significant.

## 4 Discussion

This study investigated cortical oscillatory activity associated with procedural learning in children with and without DCD. The analyses revealed that TD children learned the sequence of finger movements, evidenced by a significant increase in reaction times from the final sequence block to the subsequent random block (Janacsek and Nemeth 2013; Lum and Kidd 2012; Robertson 2007). Importantly, this behavioural learning was accompanied by expected modulations in cortical power: executing the sequence of finger movements was associated with lower beta and theta/alpha power compared to random movements. Our findings further showed that altered beta activity was associated with poorer procedural learning in DCD. In this group, the increase in reaction times from Block 3 to Block 4 was significantly smaller compared to the TD group, suggesting reduced sensitivity to the sequence. Additionally, as predicted, the DCD group exhibited an attenuated increase in beta power from sequence to random blocks, relative to TD children. Theta/alpha activity was also sensitive to sequence learning effects in both groups; however, no significant group differences were observed in these frequency bands. Thus, the relationship between procedural learning impairments in DCD and oscillatory activity outside the beta band remains unclear.

The data from the TD children showed that repeating a sequence of finger movements was associated with reduced beta power over the motor area contralateral to the responding hand. This reduction in beta power, or beta desynchronisation, and its specific scalp topography have also been observed in adult samples during sequence learning (Lum et al. 2024; Tzvi et al. 2016; Zhuang et al. 1997). As Engel and Fries (2010) proposed, beta desynchronisation may enable bottom‐up processing of sensory information to modify functional networks, whereas beta synchronisation may inhibit such updates. There is evidence from this current study that on the SRT task, beta desynchronisation was associated with improved learning. The correlation analyses showed that faster reaction times on the SRT task were associated with lower levels of beta power (see Figure 5). Our analyses also showed that beta activity was less responsive to the presence or absence of sequenced movements in children with DCD compared to TD children.

One limitation of the current study, however, is that it remains unclear whether atypical beta dynamics in DCD impede the encoding and storage of internal movement representations, or whether they reflect difficulties adapting movements in real‐time to task‐specific environmental structures. Future research could address this issue by using modified task designs that differentially emphasise motor program acquisition versus dynamic adaptation. For example, a SRT task with a fixed, repeating sequence could be compared to a version where the stimulus sequence changes unpredictably but follows environmental cues, minimising the need for internal sequence storage (Trofimova et al. 2020). Measuring EEG oscillatory dynamics across both conditions would allow tests of whether atypical beta activity in DCD primarily reflects disrupted program formation, or more general difficulties with real‐time coordination. If motor skill impairments in DCD primarily reflect disrupted motor program formation, atypical beta dynamics should be most evident in the fixed‐sequence condition. Alternatively, if impairments reflect problems with real‐time adaptation, atypical beta dynamics should also be observed during the condition requiring continuous adjustment to environmental cues.

The current study also identified a cluster of theta/alpha activity sensitive to sequence learning effects at electrode O2. Inspection of the scalp distribution of this modulation indicates that the effect occurred bilaterally over occipital regions (see Figure 3). We suspect that the identification of this cluster at electrode O2, but not O1, is more likely due to issues of statistical power rather than a restriction of theta/alpha modulation to the right hemisphere. Power from this cluster was lower on the sequence, compared to the random block and this effect was present in both groups of children (See Figure 4). Increases in theta and alpha power typically indicate greater top‐down processing of sensory information, for example, to maintain attention, or inhibit a response to irrelevant stimuli (Bonnefond and Jensen 2012; Clayton et al. 2015; Nigbur et al. 2011). On the SRT task, lower levels of theta/alpha power likely reflect disengagement of top‐down attentional oversight on sequence trials, as responses to the visual stimulus become increasingly automated (Tzvi et al. 2016). In the current study, this top‐down disengagement, however, appears to be more closely associated with tracking the visual stimulus, rather than generating manual responses, since the cluster was only identified at an occipital electrode. Thus, our results from the theta/alpha cluster likely indicate that in children, procedural learning on the SRT task reduces visuo‐spatial attentional demands, a result which mirrors research undertaken with adults (Kóbor et al. 2018; Lum et al. 2019; Tzvi et al. 2016). We acknowledge that, since sequence learning differences between the groups in the theta/alpha band were non‐significant, it remains unclear whether oscillatory activity in these frequencies is related to procedural learning problems in DCD.

## 5 Conclusion

This study showed that procedural learning of a motor sequence modulates neural oscillatory power in the theta, alpha and beta bands among children. However, beta activity over the motor area appears particularly relevant to this type of learning. In typically developing children, decreases in beta power probably encode the sequence, while increases may protect the newly acquired sequence from interference. In children with DCD, beta oscillations were less responsive to sequenced and random finger movements. Thus, atypical beta activity could be part of the neural mechanism underlying poor procedural learning in DCD. Other aspects of oscillatory activity may be intact in the disorder, during this type of learning. Theta/alpha modulation, which was associated with visuo‐spatial processing, was similarly responsive to both sequence and random trials in both the DCD and TD groups.

## Author Contributions

**Jarrad A. G. Lum**: conceptualization, funding acquisition, investigation, writing – original draft, methodology, visualization, writing – review and editing, formal analysis, data curation. **Kaila Hamilton**: writing – review and editing, project administration, supervision. **Li‐Ann Leow**: writing – review and editing, resources. **Welber Marinovic**: writing – review and editing, resources. **Ian Fuelscher**: funding acquisition, writing – review and editing, resources, supervision. **Pamela Barhoun**: writing – review and editing, resources, project administration. **Talitha C. Ford**: writing – review and editing, resources. **Aron Hill**: writing – review and editing, resources. **Samaneh Nahravani**: writing – review and editing, resources. **Melissa Kirkovski**: writing – review and editing, resources. **Peter G. Enticott**: writing – review and editing, funding acquisition, investigation, methodology, resources. **Christian Hyde**: conceptualization, investigation, funding acquisition, writing – review and editing, resources, supervision, project administration, methodology.

## Conflicts of Interest

The authors declare no conflicts of interest.

## Supporting information



Supporting information

## Data Availability

The authors have nothing to report.
