# Supplementary material for: Atypical Beta Oscillatory Dynamics Are Related to Poor Procedural Learning in Children With Developmental Coordination Disorder
Source: Dev Sci. 2025 May 21;28(4):e70031. doi: 10.1111/desc.70031 (PMC12096045; doi:10.1111/desc.70031)
Supplement: Supplementary file 1 — Supporting information [file DESC-28-e70031-s001.docx]

Supplementary Materials

**Table S1.** Correlations between oscillatory power and reaction times reported for (1) all children, (2) children with DCD and (3) TD children. *p*-values in each matric (*n* = 3) corrected using Benjamini and Hochberg (1995) false discovery rate procedure.

| **Correlation: Combined DCD & TD Groups** | | | |
| --- | --- | --- | --- |
| Variable | Avg. RT | C3 | O2 |
| 1. Avg. RT | — |  |  |
| 2. C3 | .393* | — |  |
| 3. O2 | .251 | .181 | — |
| **Correlation: DCD Group** | | |  |
| Variable | F4 | C3 | O2 |
| 1. F4 | — |  |  |
| 2. C3 | .295 | — |  |
| 3. O2 | .089 | .132 | — |
| **Correlation: TD Group** | |  |  |
| Variable | F4 | C3 | O2 |
| 1. F4 | — |  |  |
| 2. C3 | .421* | — |  |
| 3. O12 | .273 | .224 | — |

** p*_FDR Corrected_ < .05;
